# Physical Performance and Physical Activity in Older Adults: Associated but Separate Domains of Physical Function in Old Age

**DOI:** 10.1371/journal.pone.0144048

**Published:** 2015-12-02

**Authors:** Rob C. van Lummel, Stefan Walgaard, Mirjam Pijnappels, Petra J. M. Elders, Judith Garcia-Aymerich, Jaap H. van Dieën, Peter J. Beek

**Affiliations:** 1 McRoberts BV, Raamweg 43, 2596 HN The Hague, The Netherlands; 2 The Hague University of Applied Sciences, Movement Technology, The Hague, The Netherlands; 3 MOVE Research Institute Amsterdam, Department of Human Movement Sciences, Vrije Universiteit Amsterdam, Amsterdam, The Netherlands; 4 EMGO+, VU University Medical Center, Amsterdam, The Netherlands; 5 Centre for Research in Environmental Epidemiology (CREAL), Barcelona, Spain; 6 CIBER Epidemiología y Salud Pública (CIBERESP), Barcelona, Spain; 7 Universitat Pompeu Fabra, Departament de Ciències Experimentals i de la Salut, Barcelona, Spain; Texas Tech University Health Science Centers, UNITED STATES

## Abstract

**Background:**

Physical function is a crucial factor in the prevention and treatment of health conditions in older adults and is usually measured objectively with physical performance tests and/or physical activity monitoring.

**Objective:**

To examine whether 1) physical performance (PP) and physical activity (PA) constitute separate domains of physical function; 2) differentiation of PA classes is more informative than overall PA.

**Design:**

Cross-sectional study to explore the relationships within and among PP and PA measures.

**Methods:**

In 49 older participants (83±7 years; M±SD), performance-based tests were conducted and PA was measured for one week. Activity monitor data were reduced in terms of duration, periods, and mean duration of periods of lying, sitting, standing and locomotion. The relation between and within PP scores and PA outcomes were analysed using rank order correlation and factor analysis.

**Results:**

Factor structure after varimax rotation revealed two orthogonal factors explaining 78% of the variance in the data: one comprising all PA variables and one comprising all PP variables. PP scores correlated moderately with PA in daily life. Differentiation of activity types and quantification of their duration, intensity and frequency of occurrence provided stronger associations with PP, as compared to a single measure of acceleration expressing overall PA.

**Limitations:**

For independent validation, the conclusions about the validity of the presented conceptual framework and its clinical implications need to be confirmed in other studies.

**Conclusions:**

PP and PA represent associated but separate domains of physical function, suggesting that an improvement of PP does not automatically imply an increase of PA, i.e. a change to a more active lifestyle. Differentiation of activity classes in the analysis of PA provides more insights into PA and its association with PP than using a single overall measure of acceleration.

## Introduction

Physical function is increasingly recognized as a powerful factor in the prevention and treatment of a number of health conditions in older adults [1]. It is defined as one's ability to carry out activities that require physical actions, ranging from self-care (activities of daily living) to more complex activities that require a combination of skills, often with a social component or within a social context [2]. Physical function is a multidimensional concept, with four related subdomains: mobility (lower extremity function), dexterity (upper extremity function), axial ability (neck and back function), and ability to carry out instrumental activities of daily living [2]. Physical function is usually measured objectively with physical performance tests [3,4] and/or physical activity monitors [5,6].

The present study focuses on mobility as measured with physical performance (PP) tests. Over the past decades, various PP tests have been developed to assess the physical function of older adults. Typical outcome measures, such as the time to perform a supervised and standardized task, are straightforward to determine and objective, and therefore widely employed. In this study we used the timed Sit-to-Stand test (STS) [7], the Timed Up and Go test (TUG) [8,9], and the Short Physical Performance Battery (SPPB) [10]. Minimal meaningfull change of the SPPB in older adults has been reported [11].

Physical activity (PA) is defined as any bodily movement produced by skeletal muscles that requires energy expenditure [12]. PA is behavior that encompasses all forms of activity, including walking and cycling, active play, work-related activity, and active recreation such as working out in a gym, dancing, gardening and competitive sports. Self-report is the most commonly used method to measure PA in large observational studies. Yet with the advent of ambulatory movement registration techniques in the early nineties, PA is increasingly being measured by means of accelerometers cached in wearable devices. Such activity monitors allow objective assessment of the intensity, frequency, and duration of physical activity [5, 13]. The level of activity is expressed in activity counts and energy expenditure estimates [14]. In recent years, multi-axis accelerometers, recording both the magnitude and direction of accelerations, have become available, allowing detection of the orientation of the instrumented segment in question (e.g., the trunk) relative to gravity. Based on this feature, analysis methods have been developed to differentiate activities like sitting, standing, lying and locomotion [15]. We are unaware of any publications discussing the meaningful change of physical activity using activity monitors.

PP and PA are often used as outcome variables in (clinical) studies on effects of preventive or curative interventions aimed at improving physical function. In a recent review it was concluded that only limited evidence exists to support the effectiveness of pulmonary rehabilitation and pharmacotherapy in improving PA in Chronic Obstructive Pulmonary Disease (COPD) [16].

**Fig 1:**
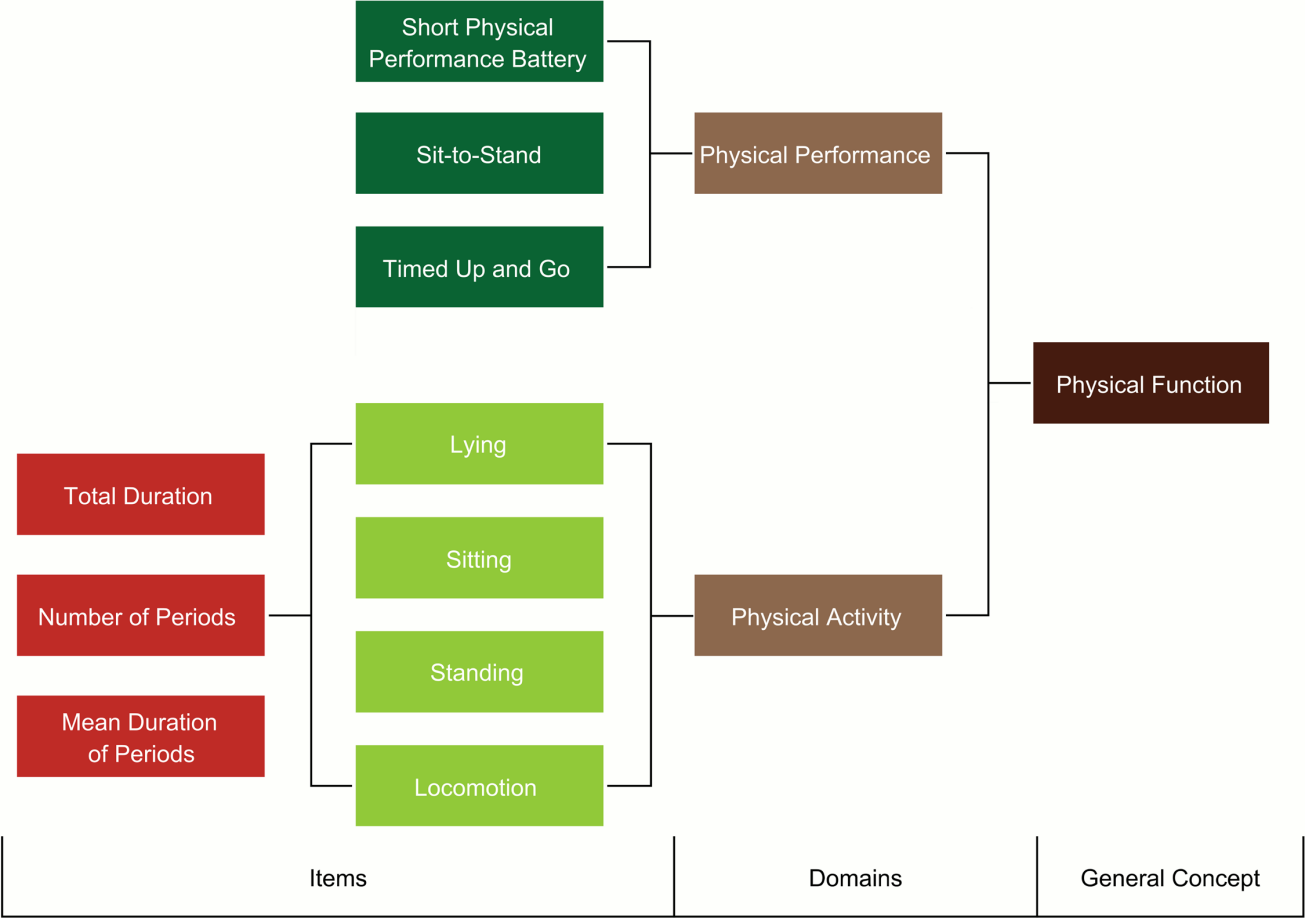
Mobility measures presented in a framework with physical performance and physical activity as domains of physical function. Activity classes are determined and for all types of physical activity total duration, number of periods and mean duration of periods are calculated.

For measurements of patient reported outcome (PRO) endpoints, the Food and Drug Administration (FDA) recommends the use of appropriate conceptual frameworks, which explicitly define the concepts measured by a PRO instrument [17]. A PRO is any report of a patient’s health status that comes directly from the patient without interpretation of the patient’s response by a clinician or someone else. A systematic review of the use of patient reported measures of PA and related constructs concluded that selected instruments lacked justification in terms of such a framework [18]. Here, we propose a conceptual framework in which PP and PA represent associated but also separate domains of the mobility domain of physical function (Fig 1) and hence require different types of measurement.

The relationship between physical activity of community dwelling older adults and functional limitations, disability or loss of independence has previously been reviewed [19]. However, in this review, articles reporting associations between physical performance tests and physical activity were not included. In patients with COPD and healthy controls, a strong correlation (0.76) between the distance walked during a 6-minute walking test and the total walking time in daily life has been found [20]. Similarly, better scores on the Short Physical Performance Battery (SPPB) appeared to be associated with higher PA levels and mobility in healthy older men [21], although in another study poor correlations were found between SPPB scores and time spent walking in daily life in healthy older people of both genders [22]. The correlation reported between the SPPB and activity counts was 0.48 [21] and while the correlation between the gait speed score of the SPPB and the amount walking in daily life was 0.35 [22]. These associations were relatively low and explain only the associations between PP and PA to a certain extent. In a recent study observations using a wearable device suggest that laboratory gait measurements do relate to daily-life walking, but are more indicative of an individual’s ‘best’ performance, rather than their usual performance [23]. All of the studies provided some support that PP and PA are associated, but did not explicitly state or test the assumption that PP and PA are related but separate domains of physical function. As physical performance is ability and physical activity is behaviour, it could be assumed that they are related but also separate domains. To our knowledge, associations between objective PP tests and PA measures have not been systematically studied to date.

The primary aim of this study was to test this hypothesis by investigating the correlation and the latent variables between PP and PA measures in older adults. In addition, we tested the hypothesis that differentiation of activity classes and quantification of their duration, intensity and frequency of occurrence, provide more meaningful relations with physical performance, than a single acceleration measure (e.g. counts) expressing overall PA.

## Methods

### Study population

For the purpose of this cross-sectional study, a convenience sample was recruited from both a residential care facility and the surrounding community in order to obtain sufficient variability in both PP and PA. Eligible persons were aged 70 years and older, had a Mini-Mental State Examination score [24] > 18 out of 30, and were able to walk 20 m without cardiac or respiratory symptoms. The medical ethical committee of the VU University Medical Center Amsterdam approved the protocol for the study (#2010/290), and all participants provided written informed consent.

### Physical performance assessment

Participants’ PP was measured by means of the 3×Sit-to-Stand (STS) [7], the TUG [9], and the SPPB [10]. Participants performed 3 STS cycles at a self-selected speed (start and end in a sitting position), while being free to swing their arms. A standard chair without arm rests was used. The patients started the TUG while sitting on a regular chair, with a height of 43–46 cm without armrests. Patients were instructed to sit with their back against the back of the chair, feet placed on the floor directly in front of the chair, and arms resting in their lap. Patients were instructed to rise from the chair (without using their arms) after the rater gave the starting signal, comfortably walk the clearly marked distance of 3 meter, turn around the cone, walk back to the chair and sit down with their back against the chair. The 3 meter walking distance was measured from the front of the chair to the middle of the cone. The SPPB consisted of measures of standing balance, walking speed, and ability to rise from a chair. For tests of standing balance, the subjects were asked to attempt to maintain their feet in the side-by-side, semi-tandem, and tandem positions for 10 seconds each. Walking speed was measured over a distance of 4 meters. Participants started standing still with their feet against a line. At a start signal they walked at self a chosen-speed and passed a second line at 4 meters distance and stopped at a line at 5 meters. The time of the faster of two walks was used for scoring. To test the ability to rise from a chair, a straight-backed chair was placed next to the wall; participants were asked to stand up and sit down five times as quickly as possible, and were timed from the initial sitting position to the final standing position at the end of the fifth stand. These three PP tests contained both preferred and maximum speed test variables; the 5×STS a subtest of the SPPB and the TUG were performed as fast as possible, whereas the 3×STS and gait as subtest of the SPPB were performed at preferred speed. All participants wore their regular footwear during all tests, and were allowed to use any mobility aid that they would normally use. However, the use of walkers or wheelchairs was precluded. These tests were administered by professionals with a background in kinesiology.

The protocol of the PP tests was implemented on a computer. Dedicated software allowed the test leader to send event markers with a remote control to start the measurements and store start and stop markers of the tests. The software used these markers to determine the duration of the 3xSTS and the TUG tests in seconds and calculate the SPPB scores for balance, walking speed, and chair rises. Five performance scores (from 0 to 4) were created for each SPPB test, with a score of 0 representing the inability to complete the test and 4 representing the highest level of performance [10].

### Physical activity assessment

**Fig 2:**
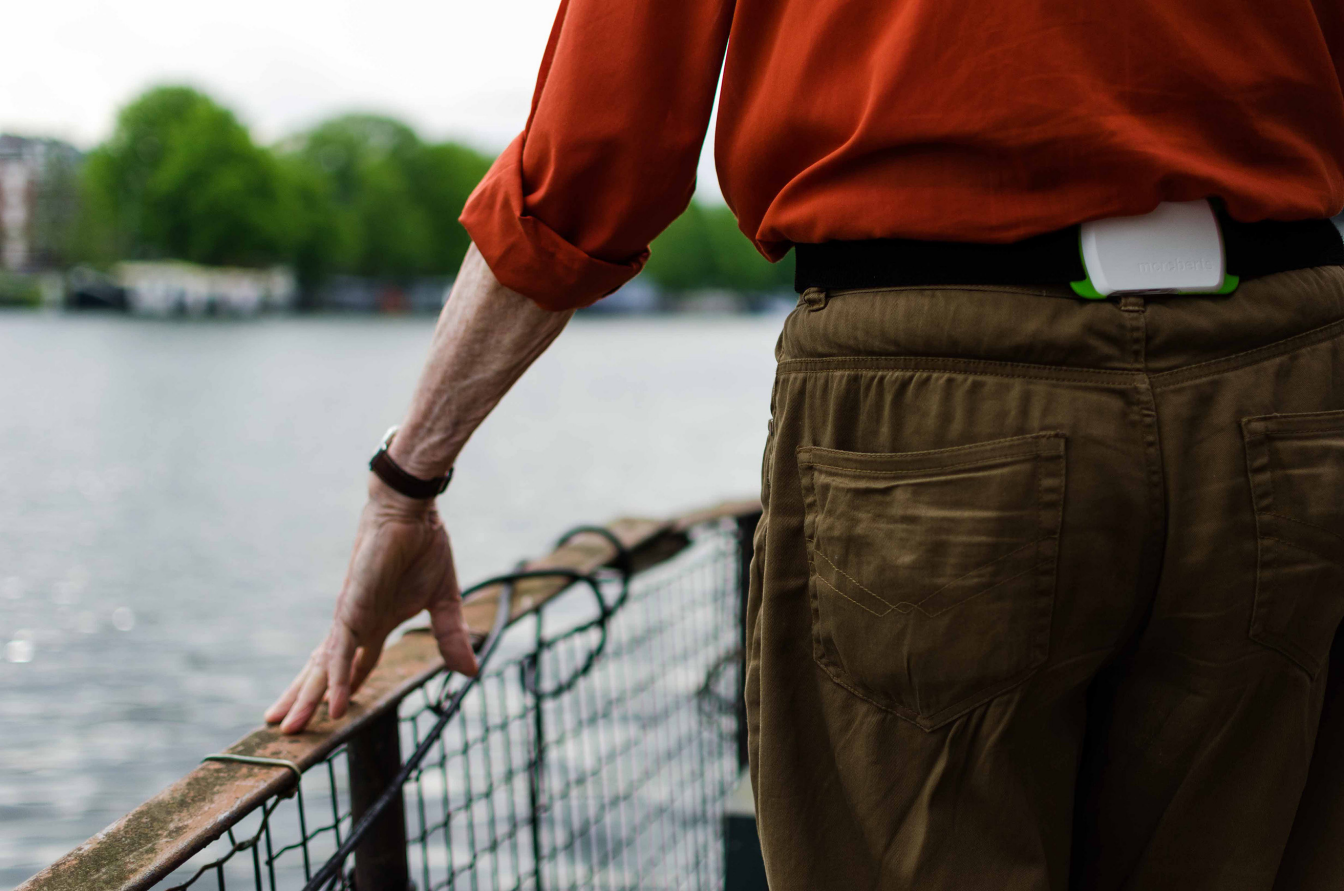
Participant wearing the activity monitor, located at the lower trunk.

PA was measured using a small and light activity monitor (51×84×8.5 mm, 45 grams), which was attached centrally over the lower back with an elastic belt around the waist (DynaPort MoveMonitor, McRoberts, The Hague, The Netherlands) (Fig 2). Participants were asked to wear the activity monitor continuously for one week (day and night) with the exception of activities involving immersion in water (e.g. showering). The monitor consisted of three orthogonal accelerometers (resolution: 0.003 g) for sensing in three directions: longitudinal (*x*), mediolateral (*y*), and anterior–posterior (*z*). Raw accelerometer signals were stored at a sample rate of 100 samples/s. Instrumental reproducibility was examined using a shaker device. Intra- and inter-instrumental intraclass correlation coefficients (ICC) were 0.99 for both *x*- and *y*-directions [13]. The direction of the z-sensor could not be tested due to a lack of space on the shaker device for solid attachment of the accelerometers. However, the sensors are expected to have the same measurement quality. The intra-instrumental coefficients of variance were smaller than 1.13% [13], indicating that reproducibility of the raw accelerometer signals was high. The validity of the activity classifications has been demonstrated in both lab [25,26] and field [27,28] studies and one week of measurement has been shown to yield highly reproducible results [29].

**Fig 3:**
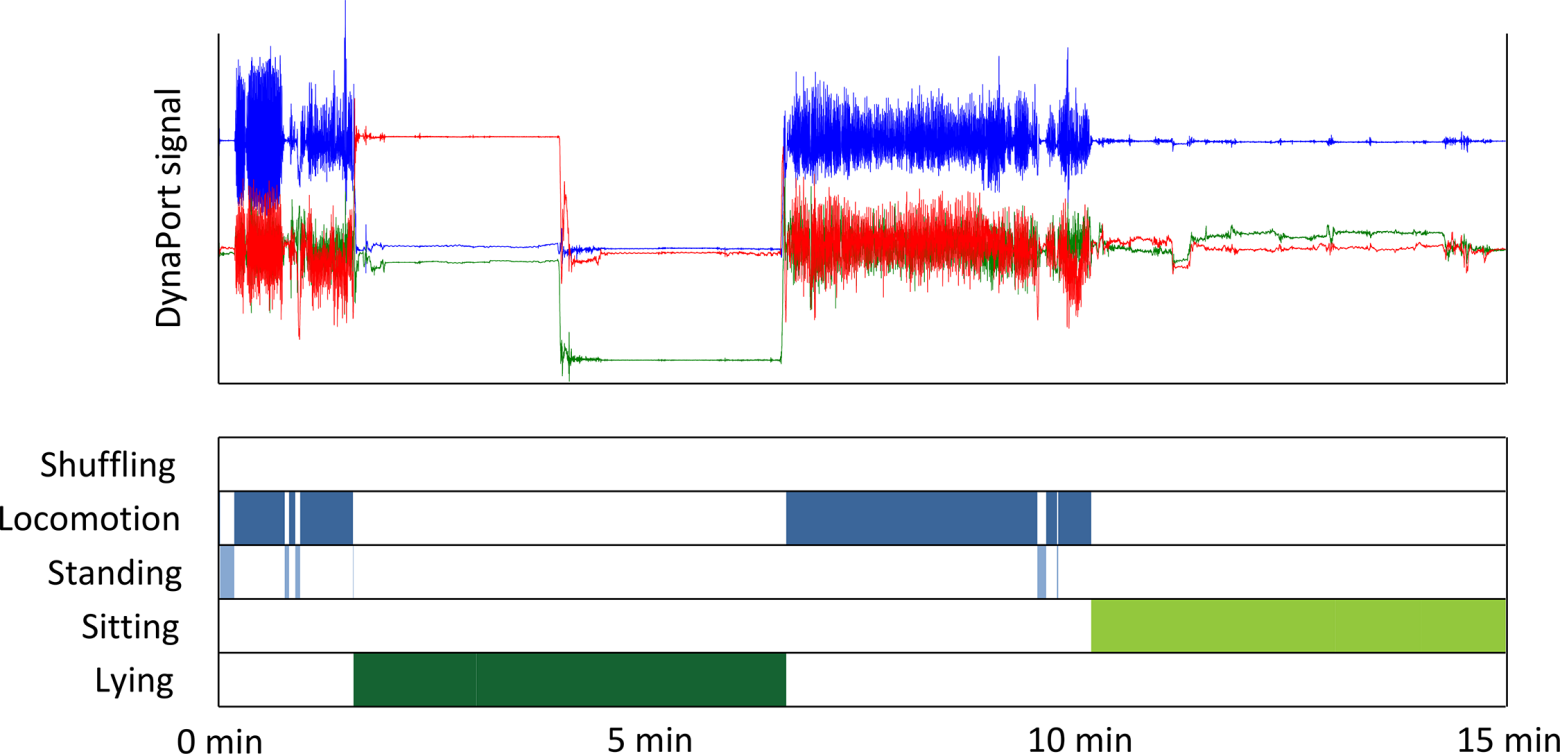
Raw acceleration signals (top panel) and a Gantt chart of classes of activity (bottom panel). The blue or dark grey line represents longitudinal (x), green or light black mediolateral (y) and red or light grey anterior-posterior (z) axis of the accelerometer. During lying, the person turns from prone to the left side.

Raw data were analysed using commercially available software (MoveMonitor, McRoberts). First, the distribution of PA classes (lying, sitting, standing, locomotion) was determined (Fig 3). Locomotion was defined broadly as all cyclic activity, including walking, stair walking and cycling. The basic ingredient of posture detection (discrimination between lying, sitting and standing) is threshold analysis on the trunk angle, as determined from the low frequency components of the accelerations. The basic ingredients of locomotion detection are threshold and frequency analysis. For each classified PA period, movement intensity (MI) was calculated. To this end, 3D accelerations were low-pass filtered to remove unwanted measurement noise and high-pass filtered to remove the effect of gravity. A fourth-order Butterworth band-pass filter was used, and cut-off frequencies were set at 0.2 and 8 Hz [13]. The Euclidean sum of the filtered 3D accelerations was used as the resultant acceleration. MI was defined as the average of the resultant acceleration during an interval and expressed in units of the acceleration due to gravity (g). Finally, for each class of activity, a week summary was made with the following variables: 1) total duration, 2) number of periods, 3) mean duration per period, and 4) weighted mean MI per period. MI was calculated per activity class and the values obtained were correlated with PP to see whether this results in more meaningful relations.

### Statistical analysis

Spearman’s rank correlations coefficients were used to explore the relationships within and between PP and PA measures. P values <0.05 were considered statistically significant. Correlations were rounded at two decimals. A correlation of <0.30 is considered as very low, a correlation between ≥0.30 to 0.50 as low, a correlation between ≥0.50 to 0.70 as moderate, and a correlation between ≥0.70 to 0.90 as high. Factor analysis (FA) was used to detect structure in and relationships between variables and to test the construct validity of the proposed conceptual framework. The fundamental objective of FA is to group together those variables that are highly correlated with each other but relatively uncorrelated with the other variables; these groups are then regarded as potential evidence for an underlying factor structure [30]. FA procedures are more accurate when each factor is represented by multiple measured variables in the analysis. PA values correlating significantly with all PP values were included in the FA. To ensure that variables had roughly normal distributions, logarithmically transformed values of PP were used. All factor analyses consisted of a principal component analysis with varimax rotation. Kaiser's eigenvalue-greater-than-one rule was applied to determine the optimal number of factors to retain [31]. A variable was assigned to a factor when its loading was at least |.50| or higher on this factor and when it had no loading at |.50| or higher on another factor. Data were analysed using SPSS 20 for Windows (SPSS Inc., Chicago, USA).

## Results

Forty-nine older participants (mean age 82.8 years (SD 6.9), 37 female, 19 residential care, 34 walking aids) were included in the study. Participant characteristics and descriptive statistics are presented in Table 1.

**Table 1:** Descriptive statistics of all measures averaged over 49 participants. Physical Performance and Physical Activity outcomes are expressed in weighted mean, standard deviation (SD), minimum and maximum values.

			Mean	SD	Min	Max
**Descriptive statistics**						
	Age	Years	82.8	6.9	70	97
	Sex	Female / Male	38 / 11	-	-	-
	Weight	Kilogram	75.4	12.3	49.1	106
	Height	Centimeter	166	8.7	149	190
	BMI	Kg / m^2^	27.4	4.4	19.4	38.1
**Physical Performance**						
		3xSTS, mean per 1xSTS (s)	1.73	.60	.9	3.5
		TUG (s)	17.9	9.44	7.5	52.4
	SPPB	Balance score	2.49	1.36	0	4
		Gait score	2.59	1.08	0	4
		5xSTS score	1.29	1.02	0	4
		Total score (Balance+Gait+5xSTS)	6.37	2.86	1	12
**Physical Activity**						
	Lying	Total duration (hours/day)	10.6	1.96	6.34	15.7
		Periods (#/day)	9.59	5.36	4.00	30.0
		Mean period duration (min/day)	82.8	40.9	22.9	227
		Movement Intensity (g)	.006	.002	.003	.014
	Sitting	Total duration (hours/day)	9.62	1.88	5.87	13.4
		Periods (#/day)	96.9	37.0	17.0	210
		Mean period duration (min/day)	7.70	5.73	2.64	28.7
		Movement Intensity (g)	.017	.006	.007	.036
	Standing	Total duration (min/day)	132	53.6	21.6	244
		Periods (#/day)	619	328	53.0	1489
		Mean period duration (s/day)	15.5	8.50	7.78	51.7
		Movement Intensity (g)	.048	.012	.025	.088
	Locomotion	Total duration (min/day)	46.1	27.6	.46	113
		Periods (#/day)	272	164	7.00	770
		Mean period duration (s/day)	10.1	3.25	3.86	21.2
		Movement Intensity (g)	.149	.028	.101	.236

Note that the sum of the 4 PA durations in Table 1 (23.24 hours) is slightly different from the mean wearing time of the sensor (23.4 hours), due to a small category of unclassified activities.

The mean duration of data collection for the 3×STS and TUG was 3.8 minutes and 7.2 minutes for the SPPB. Average wearing time of the monitor was 6.88 days with a minimum of 6 days. Mean wearing duration was 23.4 hours per day (97%).

### Are PP and PA associated?

The correlations between age, PP and PA are presented in Table 2. Age appeared only to have very low to low correlations with PP and with PA. The strength of the association between PP and PA is dependent on the activity type. Most PP outcomes significantly correlated low to moderately with 7 PA classes and very low and not significantly with 5 PA classes.

**Table 2:** Spearman rank correlations between Age, Physical Performance and Physical Activity measures. Physical performance measures includes 3xSTS, TUG, the three sub-scores and the total score of the SPPB. Physical activity scores include lying, sitting, standing and locomotion and from these total duration (Dur.), number of periods (#), mean duration of periods (Mean) and movement intensity (MI). Note that 3xSTS and TUG are expressed in seconds, where higher values indicate worse performance, whereas SPPB scores are expressed in scores of 0–4, where higher values indicate better performance.

	Age	Lying				Sitting				Standing				Locomotion				Total
		Dur.	Periods		MI	Dur.	Periods		MI	Dur.	Periods		MI	Dur.	Periods		MI	MI
			#	Mean			#	Mean			#	Mean			#	Mean		
**Age**		-.19	-.25	.19	-.07	.31	-.27	.32	-.31	-.13	-.27	.13	-.36	-.35	-.25	-.33	-.29	-.39
**3xSTS**	.29	.29	-.01	.15	-.06	.09	-.32	.33	-.21	-.29	-.40	.38	-.46	-.43	-.46	-.14	-.58	-.42
**TUG**	.27	.37	-.10	.24	.07	.22	-.53	.55	-.24	-.51	-.60	.40	-.53	-.55	-.69	-.01	-.55	-.49
**SPPB**																		
**Balance**	-.43	-.24	.14	-.21	-.06	-.17	.40	-.46	.20	.45	.51	-.22	.49	.55	.58	.24	.40	.39
**Gait**	-.36	-.31	.10	-.23	-.04	-.16	.35	-.38	.23	.37	.41	-.25	.34	.38	.48	.01	.44	.37
**5xSTS**	-.32	-.17	.06	-.12	-.02	-.30	.54	-.58	.39	.43	.62	-.50	.64	.63	.69	.11	.56	.62
**Total**	-.45	-.28	.14	-.24	-.06	-.25	.52	-.57	.32	.50	.61	-.36	.58	.61	.69	.15	.56	.54

With the exception of total duration of lying and mean duration of locomotion the significant correlations between activity classes and scores of 3xSTS performed at a self-chosen speed were markedly lower than correlations of PA with SPPB-5xSTS performed at maximum speed (Table 2).

Four PP scores (3xSTS, TUG, SPPB-Gait and SPPB-Total) showed significant low correlations (r = 0.28 to 0.37) with the total duration of lying, suggesting that participants with lower PP scores spent slightly more time lying. All PP scores correlated very low (r = -0,01 to -0.24) with the number and the mean duration of lying periods.

PP scores had very low to low correlations with total duration of sitting (r = 0.09 to -0.30). PP scores correlated low or moderately with the number and mean duration of sitting periods (r = -0.32 to -0.58). This indicates that participants with higher PP scores had more frequent sitting periods but of shorter duration.

PP scores showed low to moderate associations with the total duration and number of periods of standing and locomotion (r = -0.29 to 0.69), indicating that participants with higher PP scores stood and walked more often with more frequent interruptions.

PP scores showed very low correlations (r = -0.01 to 0.24) with mean duration of locomotion periods.

The correlations within the PA and the PP scores are presented in the online supplementary S3 Table and S4 Table.

### Are PP and PA separate domains?

The factor structure after varimax rotation revealed 2 factors (Table 3). Factor loadings for PP outcomes were low or moderate for factor 1 (0.147 to 0.465) and moderate or high for factor 2 (.590 to .846). Factor loadings for PA outcomes were high for factor 1 (0.843 to 0.925) and low for factor 2 (0.270 to 0.333). Factor 1 (PA) explained 48.8% of the variance and factor 2 (PP) explained 29.6% of the variance, adding up to a total of 78.4%.

**Table 3:** Rotated component matrix using varimax rotation displaying the factor loadings of each variable on each factor. The physical performance parameters include the duration of 3xSTS at self chosen speed, duration of he TUG and the three sub scores of the SPPB. The physical activity parameters include the number of sitting periods, the mean duration of the sitting periods, the total duration of standing, the number of standing periods, the total duration of locomotion and the number of locomotion periods. Note that 3xSTS and TUG are expressed in seconds, where higher values indicate worse performance, whereas SPPB scores are expressed in scores of 0–4, where higher values indicate better performance.

	Factor 1	Factor 2
**Physical Performance**		
3xSTS	-0.187	-0,782
TUG	-0.357	-0.824
SPPB BALANCE	0.465	0.590
SPPB GAIT	0.147	0.846
SPPB 5xSTS	0.438	0.609
**Physical Activity**		
Sitting periods	0.899	0.270
Sitting mean period duration	-0.875	-0.276
Standing total dur.	0.843	0.333
Standing periods	0.925	0.312
Locomotion total duration	0.901	0.277
Locomotion periods	0.908	0.304
% variance explained	48.8	29.6

### Is PP associated differently with the movement intensity of activity classes?

**Fig 4:**
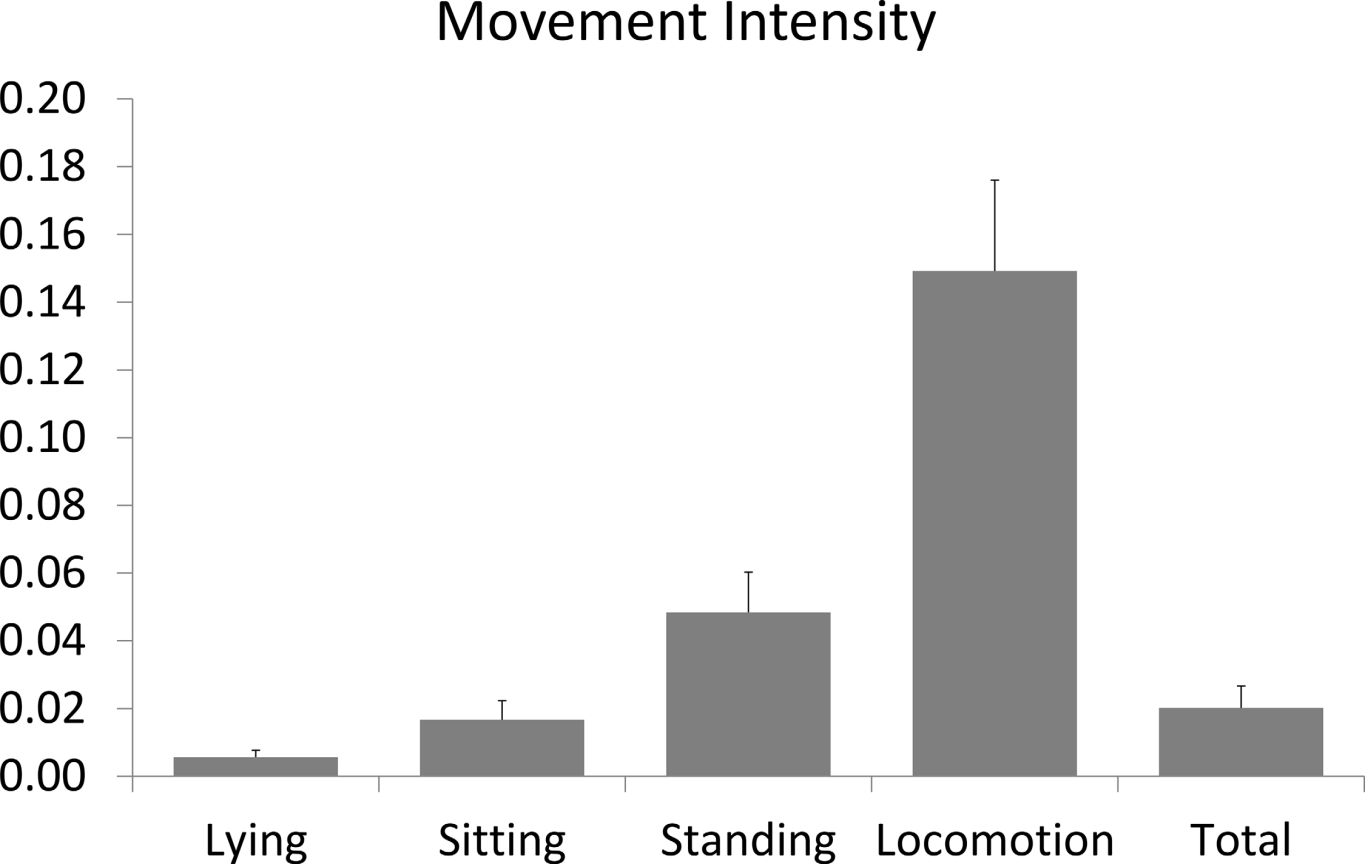
Mean Movement Intensity and standard deviations per class of activity. Differences between classes of activity were all significant (P < 0.01).

The means of movement intensity (MI) for each class of activity are presented in Fig 4. The mean MIs of the sedentary activities lying (0.006 m/s^2^) and sitting (0.017 m/s^2^) were lower than the mean MIs of the more active classes standing (0.048 m/s^2^) and locomotion (0.149 m/s^2^). The weighted mean MI over all activities classes (0.020 m/s^2^) was close to the inactive classes, because these had a much longer total duration than standing and locomotion. The PP scores correlated markedly differently with the means of the MI per activity class: lying (0.02 to -0.07), sitting (0.20 to 0.39), standing (0.34 to 0.64), and locomotion (0.29 to -0.58).

## Discussion

We aimed to validate a conceptual framework in which PP and PA constitute related but separate domains of physical function. To test this hypothesis we investigated the associations between objective PP and PA measures in older adults using rank order correlation and factor analysis. We also investigated the hypothesis that multiple different measures of PA provide more meaningful relations with PP, than a single intensity measure expressing overall PA.

### Are PP and PA associated?

In line with previous studies [5,21,22,23], we found clear correlations between PP measured with a range of performance tests and PA in daily life. Given the cross-sectional nature of the study, causation cannot be inferred and in fact causality may be circular in this case.

It was expected that sedentary activity classes (lying and sitting) would not correlate with PP, but the number and mean duration of sitting periods correlated somewhat higher than expected (0.32 to 0.58). With respect to the activity classes distinguished in the present study, the high and negative loadings of the mean durations of sitting periods (-0.875) on the PA factor (Table 3) seem to indicate that long mean durations of sitting periods are indicative of inactivity. The high positive factor loadings for the number of standing (0.925) and locomotion periods (0.908) might suggested that these measures are indicative of an active life style. Relatedly, the number of locomotion and standing episodes were generally associated moderately to high with PP (Table 2). Nicolai and co-workers [22] also found a positive correlation between SPPB and total walking time in community living older adults, albeit lower than in our study (0.41 versus 0.61).

Sitting and lying showed a more complex pattern of correlations with PP. Overall, the participants with lower PP scores showed larger total durations spent lying down and longer mean durations of sitting episodes, suggesting a less active lifestyle in the less physically fit participants, which corresponds with the lower overall MI. These findings are consistent with Healy’s [32] findings on the deleterious associations of prolonged sedentary time with cardio-metabolic and inflammatory biomarkers. Interrupting sitting time with short bouts of light or moderate-intensity walking lowers postprandial glucose and insulin levels in overweight adults. Breaking up sedentary time may be beneficial to reduce cardiovascular disease risk [33].

Remarkably, mean duration of walking periods did not correlate with the PP scores. This could be due to the low between-subject variance (3.2 s) of the mean duration (10.2 s) of walking periods, or to the fact that walking duration was predominantly determined by in-house distances.

An important finding was that PP scores from tests performed at a self-chosen pace correlated less with PA in daily life than scores from tests performed at maximum speed (Table 2). This finding is also supported by the relatively high correlations between PP tests performed at maximum speed and PA reported by Morie and co-workers [21], suggesting that PP tests performed at maximum speed more closely reflect physical capacity and skill with less interference from factors such as motivation to perform well during the test.

### Are PP and PA separate domains?

Even though PP and PA were associated, factor analysis showed that PP outcomes loaded high on one factor and low on the other factor, while PA outcomes had opposed (i.e. low and high) loadings on these factors except for balance, which had low loading on PA and moderate loading on PP. Factor 1 consisted of all PA variables and factor 2 comprised all PP variables. The FA procedure is accurate given that each factor is represented by multiple measured variables in the analysis. The resulting factor structure is simple and separated PP from PA measures, which confirms our hypothesis that PP and PA may be considered as separate but associated domains of physical function.

### Is PP associated differently with activity classes and the corresponding MI?

We showed that categorization and more detailed quantification of PA provides additional information on associations between PP and PA than the quantification of PA in terms of a single overall index of motor activity. Most activity monitors calculate activity counts or vector magnitude units over a period of time, usually over a fixed epoch of 15 seconds or one minute [14]. This method has the advantage of data reduction during the measurement, but the disadvantage that it does not allow one to differentiate classes of PA and calculate specific metrics per activity. The activity classes differed substantially in the level of activity as reflected in the MI and the total time, number of periods, and the mean duration per class of activity correlated differently with PP outcomes (Table 2). Therefore, it can be concluded that activity classification has added value over calculation of a single measure to assess physical activity.

MI as presented here expresses the weighted mean acceleration over short timeframes. The sample rate of 100 samples/s enables one to analyse a class of activity using the start and end of such an activity and calculate duration and MI per event in a very precise manner. This might be especially relevant for activity classes with short mean durations, such as the short periods of standing and locomotion in this study, with a mean duration of 15 and 10 seconds, respectively. As the correlations between PP and the MI per activity class were markedly different we can conclude that identification of activity classes reveals more specific associations between PP and PA, which remain hidden if only movement intensity is calculated.

### Practical implications

The limited correlations between PP and PA revealed by the factor analysis suggest that an improvement of PP does not automatically lead to an increase of PA, i.e. a change to a more active lifestyle. This is supported by several studies on pulmonary rehabilitation showing that translating gains in exercise capacity to increased physical activity had mixed results [16]. This has led to the implementation of physical activity interventions as part of pulmonary rehabilitation [34]. Increasing activity levels may improve long-term outcomes. It is well known that it is difficult to change from an inactive life style to a more active life style. It is common practice in interventions aimed at improving physical function to focus on PP, while it is not clear at this point whether subjects undergoing such interventions will adopt a more active life style that could affect daily life in the long-term. Having the capacity to perform mobility related physical activities does not guarantee that this capacity is actually used. There is an important role for interventions aimed at increasing physical activity. Therefore, PA measurement could be used to give objective, specific and comprehensible feedback to patients about their physical activity level in clinical practice.

### Strengths and limitations

We included a wide range of participants in this study, from normal to obese persons (BMI range 19.4 to 38.1), with ages ranging from 70 to 97, and individuals that were practically immobile (locomotion 0.46 min/day) to fairly mobile (locomotion 113 min/day). In general this heterogeneity represents a positive aspect of this study, however, it raises some concerns from a statistical point of view, given that extreme data points can have a strong effect on analyses based on correlation. We have examined this potential influence by inspecting the scatter plots of PP and PA variables. There were three outliers of inactive participants with very short walking durations, due to frequent use of a wheelchair. To evaluate the effects of these outliers we performed an additional factor analysis without these 3 outliers (S1 Table of the online supplement). This comparison revealed only a small effect of these outliers on the strength and the distribution of PP and PA factor loadings.

A potential weakness of the study is that the use of walking aids (e.g. walkers and wheelchairs) was precluded during the performance tests, while these walking aids were frequently used in daily life. This could have influenced the performance of participants with and without walking aids differentially. To examine this possibility, an additional factor analysis was performed on both groups. The distribution and the factor loadings of the PP and PA variables over the two factors hardly changed compared to the initial analysis (results presented in S2 Table of the online supplement).

Another limitation of the study is the well-known inability of accelerometers to accurately detect stationary activities, to estimate physical load associated with carrying weights, and to correct for locomotion intensity on stairs and slopes. Additionally, due to a lack of waterproofing, the monitor could not be worn during water-based activities. So our study may have underestimated PA somewhat, but given our strict criteria regarding wearing time this effect was probably small.

The results of this study are based on an older population consisting of community dwelling as well as institutionalised participants who were not selected on the basis of a specific pathology. The results therefore cannot be generalized to other populations. Further studies should provide for example subjects of younger age, other geographic areas and other chronic diseases.

Exploratory factor analysis (EFA) as applied here has its limitations. Confirmatory factor analysis (CFA) which tests the goodness of fit of a pre-specified factor model, is considered to be better suited for construct validation, because it enables testing of adequacy of fit on the data to the postulated underlying construct [31]. However, the resulting factor structure exactly matched with the structure hypothesized in our conceptual framework. For independent validation, the conclusions about the validity of the presented conceptual framework and its clinical implications need to be confirmed in other studies.

## Conclusions

Our results support a conceptual framework in which physical performance and physical activity are viewed as associated but separate domains of physical function. Activity monitors that allow differentiation of activity classes in the analysis of PA are providing new insights into PA and its association with PP.

## Supporting Information

S1 Table Rotated component matrix using varimax rotation displaying the factor loadings of each variable on each factor. The left panel shows the results of all subjects. The right panel shows the results without 3 outliers with very short total locomotion duration.
(DOCX)

S2 Table Rotated component matrix using varimax rotation displaying the factor loadings of each variable on each factor. The left panel shows the results of subjects who did not use walking aids. The right panel shows the results for subjects who did use walking aids.
(DOCX)

S3 Table Spearman rank correlations and significance between physical activity measures. Physical activity scores include lying, sitting, standing and locomotion and from these total duration, number of periods, mean duration of periods and movement intensity.
(DOCX)

S4 Table Spearman rank correlations between physical performance measures. Physical performance measures includes 3xSTS, TUG, the three sub-scores and the total score of the SPPB.
(DOCX)
