# Supplementary material for: Physical Performance and Physical Activity in Older Adults: Associated but Separate Domains of Physical Function in Old Age
Source: PLoS One. 2015 Dec 2;10(12):e0144048. doi: 10.1371/journal.pone.0144048 (PMC4667847; doi:10.1371/journal.pone.0144048)
Supplement: S2 Table — The left panel shows the results of subjects who did not use walking aids. The right panel shows the results for subjects who did use walking aids. (DOCX) [file pone.0144048.s002.docx]

**Table S2.** Rotated component matrix using varimax rotation displaying the factor loadings of each variable on each factor. The left panel shows the results of subjects who did not use walking aids. The right panel shows the results for subjects who did use walking aids. The physical performance parameters include the duration of 3xSTS in self-chosen speed, duration of he TUG and the three sub scores of the SPPB. The physical activity parameters include the number of sitting periods, the mean duration of the sitting periods, the total duration of standing, the number of standing periods, the total duration of locomotion and the number of locomotion periods.
