# Supplementary material for: Physical Performance and Physical Activity in Older Adults: Associated but Separate Domains of Physical Function in Old Age
Source: PLoS One. 2015 Dec 2;10(12):e0144048. doi: 10.1371/journal.pone.0144048 (PMC4667847; doi:10.1371/journal.pone.0144048)
Supplement: S3 Table — Physical activity scores include lying, sitting, standing and locomotion and from these total duration, number of periods, mean duration of periods and movement intensity. (DOCX) [file pone.0144048.s003.docx]

**Table S3.** Spearman rank correlations and significance between physical activity measures. Physical activity scores include lying, sitting, standing and locomotion and from these total duration, number of periods, mean duration of periods and movement intensity.

|  |  |  | **PHYSICAL ACTIVITY** | | | | | | | | | | |
| --- | --- | --- | --- | --- | --- | --- | --- | --- | --- | --- | --- | --- | --- |
| N = 49 | | |  | | **Sitting** | | | **Standing** | | | **Locomotion** | | |
|  |  |  | Periods | | Total duration | Periods | | Total duration | Periods | | Total duration | Periods | |
| **PHYSICAL ACTIVITY** | | | Number | Mean duration |  | Number | Mean duration |  | Number | Mean duration |  | Number | Mean duration |
| **Lying** | Total duration | | 0.417 | -0.075 | -0.645 | -0.121 | -0.082 | -0.317 | -0.296 | 0.137 | -0.231 | -0.276 | -0.030 |
|  |  |  | 0.003 | 0.610 | 0.000 | 0.406 | 0.575 | 0.026 | 0.039 | 0.347 | 0.110 | 0.055 | 0.838 |
|  | Periods | Number |  | -0.913 | -0.410 | 0.277 | -0.408 | 0.079 | 0.018 | 0.176 | -0.110 | -0.018 | -0.154 |
|  |  |  |  | 0.000 | 0.003 | 0.054 | 0.004 | 0.590 | 0.903 | 0.225 | 0.451 | 0.903 | 0.292 |
|  |  | Mean duration |  |  | 0.240 | -0.351 | 0.416 | -0.219 | -0.125 | -0.121 | 0.068 | -0.073 | 0.190 |
|  |  |  |  |  | 0.097 | 0.014 | 0.003 | 0.130 | 0.391 | 0.406 | 0.640 | 0.616 | 0.190 |
| **Sitting** | Total duration | |  |  |  | -0.372 | 0.614 | -0.205 | -0.257 | 0.257 | -0.301 | -0.279 | -0.157 |
|  |  |  |  |  |  | 0.009 | 0.000 | 0.158 | 0.075 | 0.074 | 0.035 | 0.052 | 0.281 |
|  | Periods | Number |  |  |  |  | -0.936 | 0.800 | 0.780 | -0.347 | 0.686 | 0.764 | 0.093 |
|  |  |  |  |  |  |  | 0.000 | 0.000 | 0.000 | 0.015 | 0.000 | 0.000 | 0.523 |
|  |  | Mean duration |  |  |  |  |  | -0.742 | -0.749 | 0.386 | -0.697 | -0.741 | -0.170 |
|  |  |  |  |  |  |  |  | 0.000 | 0.000 | 0.006 | 0.000 | 0.000 | 0.244 |
| **Standing** | Total duration | |  |  |  |  |  |  | 0.743 | -0.141 | 0.685 | 0.761 | 0.129 |
|  |  |  |  |  |  |  |  |  | 0.000 | 0.334 | 0.000 | 0.000 | 0.378 |
|  | Periods | Number |  |  |  |  |  |  |  | -0.694 | 0.888 | 0.934 | 0.153 |
|  |  |  |  |  |  |  |  |  |  | 0.000 | 0.000 | 0.000 | 0.294 |
|  |  | Mean duration |  |  |  |  |  |  |  |  | -0.641 | -0.619 | -0.171 |
|  |  |  |  |  |  |  |  |  |  |  | 0.000 | 0.000 | 0.240 |
| **Locomotion** | Total duration | |  |  |  |  |  |  |  |  |  | 0.869 | 0.449 |
|  |  |  |  |  |  |  |  |  |  |  |  | 0.000 | 0.001 |
|  | Periods | Number |  |  |  |  |  |  |  |  |  |  | 0.065 |
|  |  |  |  |  |  |  |  |  |  |  |  |  | 0.657 |

Correlations of ≥ 0.3 and < 0.5 are highlighted in light grey, correlations of ≥ 0.5 are highlighted in grey
