# Supplementary material for: Physical Performance and Physical Activity in Older Adults: Associated but Separate Domains of Physical Function in Old Age
Source: PLoS One. 2015 Dec 2;10(12):e0144048. doi: 10.1371/journal.pone.0144048 (PMC4667847; doi:10.1371/journal.pone.0144048)
Supplement: S4 Table — Physical performance measures includes 3xSTS, TUG, the three sub-scores and the total score of the SPPB. (DOCX) [file pone.0144048.s004.docx]

**Table S4.** Spearman rank correlations between physical performance measures. Physical performance measures includes 3xSTS, TUG, the three sub-scores and the total score of the SPPB.

|  | **PHYSICAL FUNCTION** | | | | |
| --- | --- | --- | --- | --- | --- |
| N = 49 | TUG | SPPB BALANCE | SPPB GAIT | SPPB 5xSTS | SPPB TOTAL |
| **PHYSICAL FUNCTION** |  |  |  |  |  |
| 3xSTS | 0.601 | -0.385 | -0.576 | -0.546 | -0.604 |
|  | 0.000 | 0.006 | 0.000 | 0.000 | 0.000 |
| TUG |  | -0.669 | -0.810 | -0.630 | -0.843 |
|  |  | 0.000 | 0.000 | 0.000 | 0.000 |
| SPPB BALANCE |  |  | 0.466 | 0.606 | 0.866 |
|  |  |  | 0.001 | 0.000 | 0.000 |
| SPPB GAIT |  |  |  | 0.500 | 0.765 |
|  |  |  |  | 0.000 | 0.000 |
| SPPB 5xSTS |  |  |  |  | 0.833 |
|  |  |  |  |  | 0.000 |

Correlations of ≥ 0.3 and < 0.5 are highlighted in light grey, correlations of ≥ 0.5 are highlighted in grey
